# Social Media Exposure and Dietary Quality in University Restaurant Consumers: A PLS-SEM Approach from Northern Peru

**DOI:** 10.3390/foods15132360

**Published:** 2026-07-02

**Authors:** Luis Edgardo Cruz Salinas, Marco Agustín Arbulú Ballesteros, Marilú Trinidad Flores Lezama, Carlos José Sandoval Reyes

**Affiliations:** Institute for Research in Science and Technology, César Vallejo University, Campus Chepén, Trujillo 13001, Peru; lcruzs@ucv.edu.pe (L.E.C.S.); mfloresl@ucv.edu.pe (M.T.F.L.); cjsandovalr@ucvvirtual.edu.pe (C.J.S.R.)

**Keywords:** food choices, dietary quality, social media, TikTok, PLS-SEM, KIDMED

## Abstract

University students face dietary transitions shaped by time constraints, campus food environments, and intensive exposure to food-related content on social media, yet the mechanisms linking digital exposure to observable food choices and overall diet quality remain insufficiently modeled in Latin American contexts. This study examined whether social media-driven food norms (NI) and in-restaurant food choices (CD) sequentially mediate the effect of Instagram (IG) and TikTok (TK) exposure on overall diet quality (Y), while incorporating physical activity (PA) as an independent predictor. This is a quantitative cross-sectional study based on a paper questionnaire administered face-to-face to 615 university students (53.2% women; M = 21.7 years; 39.2% public, 60.8% private universities) eating in campus restaurants in La Libertad, northern Peru. Data were analyzed through PLS-SEM (SmartPLS 4) with 5000 bootstrap resamples and BCa 95% confidence intervals; Y was operationalized through a culturally adapted KIDMED index. All five structural hypotheses were supported: TK → NI (β = 0.479) exceeded IG → NI (β = 0.349); NI → CD (β = 0.473) and PA → CD (β = 0.216) operated as independent pathways; and CD → Y (β = 0.255) confirmed the distal link. NI fully mediated both digital pathways toward food choices. Diet quality in university restaurants is reconfigured primarily through normative, not informational, digital mechanisms, suggesting norm-based interventions over nutrition-information campaigns. Reflective measures showed adequate internal consistency for IG, TK, and NI (Cronbach’s α = 0.874–0.889; CR = 0.907–0.923) and convergent validity (AVE = 0.620–0.751); the structural model explained 55.5% of the variance in NI, 30.8% in CD, and 34.8% in overall diet quality.

## 1. Introduction

University admission usually coincides with changes in dietary patterns that affect health, well-being and academic performance. In the Peruvian context, cross-sectional studies document mixed dietary profiles: a high frequency of fruit and vegetable consumption coexists with significant consumption of ultra-processed foods, while socioeconomic and lifestyle factors amplify nutritional risk [1,2]. Reviews of interventions targeting diet, physical activity, and weight in college students show that environmental and digital approaches tend to produce more consistent improvements in cognitive variables than in sustained behavioral changes [3], reinforcing the value of identifying mechanisms linking digital exposure to effective choice. Multicenter designs propose to simultaneously measure multiple lifestyle behaviors and their association with mental health symptoms during college life, assuming interdependence between habits [4].

In Peru, cross-sectional evidence in university students shows that dietary intake is associated with contextual and behavioral factors. In post-COVID-19 times, the consumption of food groups is related to family coexistence, the place of consumption, alcohol consumption, economic changes in the home, physical activity and sleep hours [1]. In Trujillo, university students reported a high frequency of fruit and vegetable consumption together with significant consumption of ultra-processed foods [2]. Qualitative studies in Lima on barriers and facilitators of nutrition and physical activity document obstacles such as a lack of practical information and limitations of the community environment [5].

Exposure to social media content can influence eating behaviors through changes in preferences, perceived norms, and intentions. In Peruvian university students (*n* = 311), a strong positive relationship was reported between the use of social networks and eating habits, estimated using structural equations (ρ = 0.832, *p* < 0.001) [6]. In adolescents, convenience preference mediated the negative link between network use and dietary satisfaction [7]. A specific mechanism is Food FOMO (Fear of Missing Out), which describes the social motivation to participate in digitally disseminated gastronomic trends, incorporated into the present model as a normative signal guiding perceptions and consumption choices.

The theoretical scaffolding integrates three conceptual families. The Theory of Planned Behavior (TPB) posits that behavior is preceded by intention, structured from attitudes, subjective norms, and perceived behavioral control [8]. Empirical applications show that network exposure can be related to attitudes and subjective norms in behaviors of sustainable consumption [9,10]. Recent PLS-SEM evidence in lifestyle product categories shows that social media communication shapes consumer attitudes and, through them, purchase intentions [11], reinforcing the attitudinal–normative chain modeled here for food choices. Social Learning Theory (SLT) complements the approach: in social networks, influencers and peers act as role models and digital interactions as reward signals [12]. In Peruvian evidence applied to consumption, influencers influence purchase decisions with heterogeneity according to message dimensions [13]. The Dual Processing Model (DPM) distinguishes central elaboration versus heuristic routes; in rapid content consumption, cues such as visual appeal and popularity can operate as decisional shortcuts even with low cognitive elaboration [14,15]. In food influencers on Instagram, credibility and parasocial relationships were associated with intentions to visit restaurants [16].

The links between network use and eating are not univocally benign. In Peruvian university students (*n* = 297), a significant relationship between social network addiction and eating disorders was reported (r = 0.295, *p* < 0.05) [17]. In female adolescents in Lima (*n* = 269), higher network use scores were associated with a higher risk of eating disorders after adjustment for age and BMI-for-age [18]. In Peruvian households during the COVID-19 pandemic, associations were observed between sociodemographic factors and intake of fruits, vegetables and ultra-processed foods [19]; and in regional analyses with ENAHO and NOVA classification, consumption patterns of processed foods differed between the coast, highlands and jungle [20].

The research gap is located at four levels. First, the available Peruvian evidence is concentrated in university students in specific cities (Huancayo, Lima, Trujillo) with cross-sectional designs, which limits comparability by food environment and out-of-home consumption pattern [1,2,21,22]. Second, there is little integration of simultaneous exposure to Instagram and TikTok with the chain social influence of social media → food choices → dietary quality in a single structural model where the outcome is a composite/formative index such as KIDMED [23,24]. Third, physical activity has been studied predominantly as a covariate, but its role as a direct predictor of food choices in university restaurants—independent of digital exposure—has not been explicitly modeled in northern Peru [1]. Fourth, there is a lack of integration of multiple mediations with reflective (NI, CD) and formative (Y) constructs in a PLS-SEM scheme that reflects the complete chain from exposure to dietary quality [23,24].

The contribution of this study unfolds at three levels. At the theoretical level, the combination of the TPB, TAS and MPD specifies the mechanisms by which exposure to food content on Instagram and TikTok is related to the social influence of social media on food (NI), to food choices (CD) and to overall dietary quality. At the methodological level, PLS-SEM is relevant for models with chain mediations and reflective (NI, CD) and formative (Y) constructs, with predictive estimation in models with multiple causal relationships [23,24]. At the applied level, the results are useful for institutional nutritionists, public-health communicators and those responsible for the management of university canteens in northern Peru [25].

The general objective of this study was to analyze, using PLS-SEM, the relationship between the use of Instagram and TikTok with the overall quality of the diet in university restaurant consumers in northern Peru, through the social influence of social media on food (NI) and food choices (CD), incorporating physical activity as a direct predictor [23,24]. The specific objectives align one to one with H1 (Instagram → NI), H2 (TikTok → NI), H3 (NI → CD), H4 (AF → CD) and H5 (CD → Y), and with indirect mediation effects: NI as a mediator between exposure and choices, CD as a mediator between NI and dietary quality [26,27].

The eating problem in college students is presented as a multicausal phenomenon involving the food environment, time constraints, lifestyles, and academic pressures. In reviews of interventions, environmental approaches produce changes in dietary outcomes more consistently than digital educational components, which tend to impact cognitions rather than behaviors [3]. This pattern indicates that the link between intentions and effective behavior remains a challenge, especially when the context favors quick, energy-dense options.

In Peru, factors associated with college dietary intake include variables of coexistence, the place of consumption and health habits. In a post-pandemic context, specific associations linked certain food groups with family coexistence, the place of consumption, alcohol consumption, household economic changes, physical activity and sleep hours [1]. In university students from Trujillo, a high frequency of fruit and vegetable consumption was reported along with relevant consumption of ultra-processed foods [2]. The socioeconomic component conditions availability and preference for ultra-processed foods: analysis of Peruvian families documented associations between education, income, region and consumption of fruits, vegetables and ultra-processed foods [19]. In regional analyses with ENAHO and NOVA classification, differences were reported between the coast, highlands and jungle in consumption patterns of processed foods [20].

The role of social networks as a channel of influence is observed in the preference of media for food education messages. Among university students in Lima, Instagram appears among the most mentioned media to receive educational messages from Peruvian food guides [21]. In the Peruvian Amazon, university students reported that social networks influence purchasing decisions, with Instagram predominating in commercial promotion [28]. In fast food consumers in Lima (*n* = 101), a moderate relationship between digital marketing strategy and consumer experience was reported [29]. In a Peruvian theme restaurant, the consumer profile included university students who indicated Instagram as their favorite network [30].

The influence of influencers has been studied under influencer marketing logic. In millennials in Arequipa (*n* = 404), brand recognition and the perceived veracity of the influencer contributed significantly to the purchase decision, while content value and the credibility of the influencer showed no effect on it [13]. In Peruvian university students, TikTok and Instagram influenced consumption decisions by promoting attributes of authenticity and useful information [31]. In methodological terms, psychometric properties of eating behavior instruments have been evaluated in Peru, showing the feasibility of comparative analyses [32,33]. Recent bibliometric analyses describe the growing adoption of PLS-SEM in consumer behavior and management research [34].

### 1.1. Empirical Background and Research Gap

Axis 1: consolidated evidence. In Peruvian university students in Huancayo (*n* = 311), social network use showed a strong positive relationship with eating habits (ρ = 0.832, *p* < 0.001), suggesting that greater digital exposure and interaction is accompanied by modifications in eating practices [6]. In Indonesian consumers (*n* = 130) under the TPB, network exposure and pandemic context were positively and significantly related to sustainable consumption behavior, with attitudes playing a relevant explanatory role [9]. In Korean adolescents, convenience preference fully mediated the negative link between network use and dietary satisfaction [7].

Specifically for TikTok, its short video format and algorithmic amplification showed an ability to rapidly modify food perceptions among youth. In Peruvian university students, TikTok and Instagram appear to be platforms that influence consumption decisions by promoting authenticity and useful information, with differentiated roles depending on the type of content [31]. This algorithmic mechanism of repeated and personalized exposure is consistent with the peripheral route of MPD [14]. In the digital social influence literature, trust and congruence models appear as recurrent mechanisms. In a survey with PLS-SEM (*n* = 383), influencer–follower congruence positively influenced food choices and perceived trust mediated key relationships [35]. On Instagram, credibility and parasocial relationships of food influencers influenced intention to visit restaurants, with the Blogger–subscriber relationship more determinant than superficial attributes [16].

Axis 2: active debates. The results are not uniform. In Arequipa (*n* = 404), while brand recognition and perceived truthfulness contributed to the purchase decision, content value and influencer credibility did not show significant effects [13]. In Taiwanese consumers (*n* = 418) of dietary supplements, credibility dimensions had differential effects on perceptions of nutritional value, reinforcing that the effects depend on which dimension is measured and on which object of consumption [36]. There are also results linking social networks with restaurant consumption without implying improvements in dietary quality: in Lima (*n* = 101), a moderate relationship between network use and consumer digital experience was reported [29]. In Trujillo, a positive and significant link between the perceived quality of Facebook content and purchase intention highlighted correlations with attractiveness, interactivity and relevance [37].

In contrast, evidence on health underscores potential risks. In Peruvian university students (*n* = 297), the relationship between social network addiction and risky eating attitudes (EAT-26) was statistically significant [17]. In female adolescents from Lima (*n* = 269), higher network use scores were associated with a higher risk of eating disorders, associations that were maintained after adjustment for age and BMI-for-age [18]. The need remains to integrate these links in a PLS-SEM model that simultaneously measures the chain digital exposure → social influence of social media on food → choices → dietary quality with a sample from northern Peru [1,2].

### 1.2. Theoretical Bases and Hypothesis Development

#### Model Constructs

Instagram (IG1-IG4) and TikTok (TK1-TK4) use are conceptualized as exposure to food content: the frequency and time of consumption, type of accounts followed and interactions (engagement). In Peru, differentiated roles by platform in consumption and information decisions have been reported [28,31], which justifies operationalizing them as independent constructs.

The normative influence of social media on food (NI) is grouped into six indicators: perceived social norms on healthy eating among peers (NI1), visual appeal of the food presented in networks (NI2), trust in influencers’ recommendations (NI3), intention to incorporate foods seen in networks into the diet (NI4), activation of the social norm for digital gastronomic trends (NI5) and perceived influence of digital trends on the type of restaurant or dish chosen (NI6). Under the TPB, attitudes, subjective norms, and perceived control are distinct facets of the volitional process that precedes behavior [8]; NI is specified as a reflective construct: the six items are interchangeable manifestations of a latent variable—social influence of networks on eating—that share a common origin in digital exposure.

Restaurant food choices (CD) operationalize out-of-home consumption decisions: the frequency of consumption of fruits and vegetables (CD1), lean protein or whole grains (CD2), fast food or ultra-processed food (CD3, reverse recoded), and sugary drinks or alcohol when eating out (CD4, reverse recoded). CD is specified as a reflective construct; higher values on all four indicators correspond to healthier choices [24]. Physical activity (PA) is incorporated into the model as a direct predictor of CD through a single indicator (ActFis): weekly moderate or vigorous physical activity time in minutes (abbreviated IPAQ) [38]. Overall diet quality (Y) is operationalized with the KIDMED index adapted to the Peruvian university context [39], composed of 14 dichotomous items with a theoretical range from −4 to +10; Y is a formative index, and reflective internal consistency criteria do not apply [24].

**H1:**  *Instagram exposure → social influence of social media on food (NI). Instagram exposes users to food content through Stories, Reels and influencer accounts. Under TAS, repeated observation of credible model eating behaviors activates descriptive norms and vicarious reinforcement* [12]. *Under CTP, such exposure reconfigures attitudes and subjective norms, antecedents of intention* [8]. *In Peruvian university students, Instagram has been identified as a preferred channel for educational messages about food* [21] *and as a platform guiding consumption decisions in restaurant contexts* [28]. *A positive effect is anticipated: greater exposure on Instagram is associated with greater social influence of social media on food.*

**H2:**  *TikTok exposure → social media social influence in food (NI). The short video format and algorithmic amplification of TikTok generate loops of exposure to personalized and repeated food content, reinforcing food salience and subjective norms about what one is supposed to eat. Under MPD, virality and repetition act as heuristic cues that modify preferences with low cognitive elaboration* [14,15]. *In Peruvian university students, TikTok influenced consumption decisions by promoting authenticity and useful information* [31]. *It is anticipated that the effect of TikTok on NI will be of greater magnitude than that of Instagram, given the more invasive nature of the platform’s recommendation algorithm.*

**H3:**  *Social influence of social media on food (NI) → food choices (CD). When network exposure reconfigures perceived norms, food attractiveness, influencer trust, consumption intention, and trend awareness, those cognitive-affective states translate into observable choice decisions. Under the TPB, subjective attitudes and norms determine the intention that precedes behavior* [8]. *In PLS-SEM, perceived trust measured the relationship between influencer–follower congruence and food choices* [35]. *A positive effect is anticipated: greater social influence of social media on food is associated with food choices that reflect digital trends.*

**H4:**  *Physical activity (PA) → restaurant food choices (CD). Regular practice of physical activity acts as a marker of a health-oriented lifestyle that generates behavioral consistency. In post-pandemic Peruvian university students, physical activity was associated with higher fruit consumption (p = 0.021)* [1]. *Under Self-Determination Theory (SDT; Ryan & Deci, 2000)* [40], *regular PA practice expresses intrinsic motivation toward health that generalizes to other protective behaviors, including food choices. This motivationally induced behavioral consistency mechanism predisposes one to make protective choices over menus with limited availability* [12]. *A positive effect of smaller magnitude than NI is anticipated, given that dietary and sport determinants are partially independent.*

**H5:**  *Dietary choices (CD) → overall diet quality (Y). The adapted KIDMED index records the accumulation of protective and risky behaviors over four weeks. A pattern of choices that favors fruits, vegetables and lean protein (CD1, CD2) and avoids ultra-processed and sugary drinks (CD3, CD4 recoded) should shift the formative score balance toward higher values. In Trujillo, university students showed empirical variability in both types of choices* [2], *which the KIDMED index can capture. A positive effect is anticipated, although modest, because dietary quality also depends on factors not captured in the model (economic access, available supply, habits acquired at home).*

**Figure 1:**
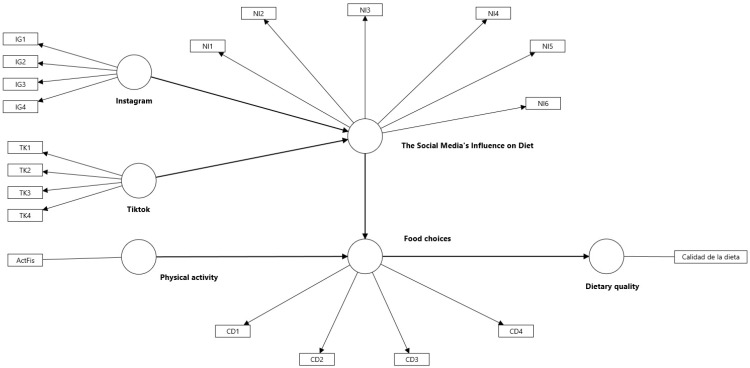
Proposed conceptual/hypothesized structural model. Hypothesized structural chain linking exposure to food-related content on Instagram (IG) and TikTok (TK) to overall diet quality (Y) through the normative influence of social media on food (NI) and restaurant food choices (CD), with physical activity (PA) modeled as an autonomous predictor of CD. Arrows denote directional hypotheses (H1–H5); reflective constructs (IG, TK, NI, CD) are estimated with PLS-SEM and Y is operationalized as a formative composite based on the culturally adapted KIDMED index.

Mediation effects: the model specifies three mediation effects. NI mediates the relationship between Instagram and food choices (IG → NI → CD): the influence of Instagram on choices operates through cognitive–attitudinal reconfiguration of NI, not directly. The same pattern applies to TikTok (TK → NI → CD). CD mediates the relationship between NI and overall dietary quality (NI → CD → Y): the social influence of social media reaches dietary quality through its behavioral expression in choices. The complete chains IG → NI → CD → CD → Y and TK → NI → CD → Y are tested as total indirect effects as summarised in Figure 1. Under the TPB, the sequence cognition → intention → behavior → behavior → outcome is theoretically consistent [8,27].

Methodological note (PLS-SEM): the specification includes chain mediations and reflective (NI, CD) and formative (Y) constructs, which justifies the use of PLS-SEM to build and test behavioral causal theory in complex models [23,24]. For mediation in PLS-SEM, contemporary procedures based on bootstrapping and an explicit discussion of the type of mediation are employed; updated criteria for the selection and interpretation of effect sizes are reported [26,27].

## 2. Materials and Methods

### 2.1. Study Design

The study is quantitative, non-experimental and cross-sectional. The unit of analysis is university students from northern Peru who regularly eat in canteens or restaurants on campus. The central purpose is to quantify—through PLS-SEM—the path coefficients between the use of Instagram (IG) and TikTok (TK), the social influence of social media on food (NI), food choices in restaurants (CD), physical activity as a direct predictor (PA) and overall diet quality (Y = adapted KIDMED index). The model contains five direct effect hypotheses (H1–H5) and three mediation effects.

The cross-sectional design restricts conclusions to theoretically supported causal plausibility, not proven causality. To mitigate this risk, the causal model is anchored in the CTP, TAS and MPD, which establish the plausible temporal direction of the effects independent of simultaneous measurement; procedural and statistical controls for common method bias were applied [41]; and robustness analyses were performed with partial respecification of the structural model.

The directional interpretation of the IG/TK → NI → CD chain is theoretically anchored in the TPB, SLT and the Dual Processing Model [8,12,14,15], which place cognitive–attitudinal reconfiguration before observable behavior. PLS-SEM is a predictive estimator [23,24], and “mediation” in the present analysis refers to indirect predictive paths in the sense of Nitzl et al. [27], not to causal mediators in the counterfactual framework. A reverse-causal interpretation, by which students with pre-existing healthy eating habits selectively follow nutrition-oriented digital content, is not implausible but is theoretically secondary in this design and unsupported in the comparable literature [6,9,35], where the same anchoring has been adopted in published cross-sectional PLS-SEM evidence on social media use and eating behavior.

### 2.2. Context, Population and Sampling

The target population comprises students with current enrollment in public and private universities in northern Peru (department of La Libertad; provinces of Trujillo, Chepén, Pacasmayo, and Ascope), who were present in the physical space of the university canteen on their campus during the second semester of 2024, regardless of whether they chose from the canteen menu or brought food of external origin. This criterion reflects the reality of the survey: the questionnaire was applied in the dining hall space to all students present there at the time of the survey. The university canteen setting is conceptually relevant because it constitutes the decision context in which CD is operationalized: faced with a limited menu, fixed prices and restricted availability, previous digital exposure can materialize in observable choices. The study covers university restaurants only; the unit of observation is the in-canteen consumer at one public and one private institution per participating province, so the design speaks to the population of campus-restaurant users in this region rather than to general food consumers in northern Peru.

Inclusion criteria required active enrollment during the survey period, presence in the university dining space during the survey period (regardless of the origin or type of food consumed) and regular use of Instagram or TikTok with consumption of food content. Participants under 17 years of age, students with prescribed therapeutic diets and questionnaires with more than 15% of omitted items were excluded.

The sampling was non-probabilistic by convenience, by direct contact at the entrances to the university canteens. The minimum sample size was determined by a priori power analysis with G*Power 3.1.9.7 (Heinrich Heine University, Düsseldorf, Germany) [42]. Under a medium effect size (f^2^ = 0.15), α = 0.05 and power 1 − β = 0.80, the minimum required was *N* = 98; with power 1 − β = 0.95, the threshold amounted to *N* = 153 [43]. Since the model includes chain mediations, the target was set to exceed 200 cases to ensure stability of the bootstrap estimates [44]. The final sample was *N* = 615, composed of students from public (39.2%) and private (60.8%) universities in the department of La Libertad, Peru.

### 2.3. Measurement of Variables and Instrumentation

The questionnaire comprises five blocks. The first four—exposure on Instagram, exposure on TikTok, social influence of social media on food and food choices in restaurants—employ five-point Likert scales (1 = never/totally disagree; 5 = always/totally agree). The fifth block, overall diet quality, is a dichotomous index. The idiomatic and cultural adaptation of the instrument followed an expert judgment procedure (*n* = 5 judges specialized in nutrition and consumer behavior) and a cognitive pilot (*n* = 30 students).

#### 2.3.1. Exposure to Food Content on Instagram (IG)

Four items (IG1–IG4) measure daily exposure time to food content, type of accounts followed, level of engagement and perceived influence of Stories/Reels on consumption decisions. Attitudinal items are anchored at 1 = strongly disagree/5 = strongly agree; frequency items are anchored at 1 = never/5 = always.

#### 2.3.2. Exposure to Food Content on TikTok (TK)

Four items (TK1–TK4) capture time of exposure to food content, influence of algorithmic feed on preferences, saving or sharing behavior of food videos, and motivational effect of viral trends on trying dishes or restaurants. The measurement logic parallels that of IG to facilitate direct comparability of IG → NI and TK → NI paths in the structural model.

#### 2.3.3. Social Influence of Social Media on Food (NI)

Six items (NI1–NI6) integrate this mediator: perceived social norms about healthy eating among peers (NI1), visual appeal of the food presented in networks (NI2), trust in influencers’ recommendations (NI3), intention to incorporate foods seen in networks into the diet (NI4), activation of a digital gastronomic trend as a social norm—perception that the social environment has already adopted certain trending foods or restaurants and implicit pressure to align (NI5)—and perceived influence of digital trends on the type of restaurant or dish chosen (NI6). NI is specified as a reflective construct: the six items are interchangeable manifestations of the social influence of networks on eating as a single latent variable, with common causal origin in digital exposure [24]. External loadings ≥ 0.70, AVE ≥ 0.50 and HTMT < 0.85 are expected.

#### 2.3.4. Restaurant Food Choices (CD)

Four items (CD1–CD4) operationalize out-of-home consumption decisions: frequency of consumption of fruits and vegetables (CD1), lean protein or whole grains (CD2), fast food or ultra-processed food (CD3), and sugar-sweetened beverages or alcohol when eating out (CD4). CD1 and CD2 are protective indicators; CD3 and CD4 are indicators of risk (expected negative loadings). CD is specified as a reflective construct: the four items are interchangeable manifestations of the food choice pattern as a latent variable, with common causal origin in the student’s preferences [24]. CD3 and CD4 were inversely recoded so that high loadings on all indicators correspond to healthier choices.

#### 2.3.5. Physical Activity (PA)

Physical activity is incorporated into the model as a direct predictor of food choices (H4), not as a moderator. It is operationalized with a single indicator (physical activity): weekly amount of moderate or vigorous physical activity self-reported in minutes, estimated using the International Physical Activity Questionnaire (IPAQ, abbreviated version) [38]. The theoretical rationale is grounded in SDT (Ryan & Deci, 2000) [40]: regular PA expresses intrinsic health-oriented motivation that generalizes to protective food choices through a process of behavioral consistency. This mechanism is more robust than operant reinforcement frameworks, as it operates from the internal agency of the learner and not from environmental contingencies [1,12]. Since it is a single indicator, its load is set at 1.0 in the PLS-SEM model and no internal consistency criteria apply.

The independence of PA from the digital pathway is a theoretical specification grounded in Self-Determination Theory [40], which predicts that intrinsically motivated behavior generalizes across domains autonomously of external social validation; an interaction between PA and digital exposure would contradict the SDT mechanism tested in the present model. To empirically validate this specification, a post hoc sensitivity analysis was performed in which the PA × IG and PA × TK product–indicator interaction terms were added to the structural equation predicting CD; the corresponding estimates are reported in Section 3.

#### 2.3.6. Overall Diet Quality (Y)

Y is operationalized with the KIDMED index [39] systematically adapted to the Peruvian university context. The adaptation replaced Mediterranean referents with local equivalents with an analogous nutritional profile: (a) generic legumes → menestras (lentils, beans, pallares), staple food of the Peruvian lunch; (b) industrial pastries → cookies with filling or ultra-processed breakfast products; (c) generic fast food → grilled chicken combos or hamburgers; (d) olive oil → avocado oil or vegetable oil in preparations. The instrument retains 14 dichotomous items (Yes/No) referring to the previous four weeks, Ten correspond to protective behaviors (+1 each) and four to risk behaviors (−1 each), with a theoretical range from −4 to +10. Given that Y is a formative index, the reflective internal consistency criteria (Cronbach’s alpha, AVE) are statistically inappropriate; the evaluation focused on the absence of multicollinearity between indicators (VIF < 3.3) and on the theoretical justification of each item [24].

### 2.4. Data Collection Procedure

The questionnaires were administered face-to-face on printed paper forms (no online surveys, no Google Forms or similar digital platforms were used) between August and November 2024 in the university canteens of the participating institutions. Trained research assistants approached students at the canteen entrance, briefly explained the study, and handed the booklet (eight pages including consent form, sociodemographic block, four Likert blocks for IG, TK, NI, CD, and the 14-item KIDMED block) to those who agreed to participate; completion took approximately 12–15 min. Participants agreed voluntarily after reading the informed consent, which guaranteed the anonymity of the responses, the absence of academic consequences for not participating, and the exclusive use of the data for research purposes. The questionnaire did not reveal the underlying causal model to reduce hypothesis-driven response bias.

Duplicate records were identified by cross-checking sociodemographic data. The pattern of missing data was assessed with Little’s test [45]; with confirmed MCAR and less than 5% missing per item, mean imputation was applied. Common method bias was managed with procedural (physical separation of KIDMED block from Likert blocks, reiteration of anonymity, randomization of item order) and statistical measures: exploratory Harman test [46], total collinearity diagnosis (VIF; threshold > 3.3) [47] and marker variable method (Lindell & Whitney, 2001) [48]. An item theoretically unrelated to the model constructs was introduced as a marker variable; correlations corrected for estimated marker variable bias remained substantially the same as the original correlations, indicating absence of relevant common method bias.

Beyond the procedural and statistical safeguards described above, the dataset met the established quality criteria for PLS-SEM analysis: (i) sample size *N* = 615 substantially exceeded the minimum of *N* = 153 required by a priori G*Power analysis under f^2^ = 0.15, α = 0.05 and 1 − β = 0.95, as well as the inverse-square-root threshold of *N* = 218 for the most complex path of the model; (ii) Little’s MCAR test confirmed missing-completely-at-random pattern with less than 5% missing per item; (iii) all complete-collinearity VIF values remained below 3.3, ruling out common method bias under Kock’s (2015) criterion [47]; (iv) Harman’s single-factor variance was 28%, well below the 40% threshold; (v) the marker variable correction (Lindell & Whitney, 2001) preserved the original correlation matrix [48]; and (vi) bootstrapping with 5000 resamples and BCa confidence intervals provided robust standard-error estimation. The data format and analytical depth therefore meet the methodological standards for inferential PLS-SEM in behavioral research [23,24].

### 2.5. Statistical Analysis Plan

The statistical analysis was run in SmartPLS 4.1.1.8 (SmartPLS GmbH, Oststeinbek, Germany) [49], with SPSS Statistics version 29.0 (IBM Corp., Armonk, NY, USA) as an auxiliary tool for initial cleaning and descriptive characterization. The preference for PLS-SEM over CB-SEM responds to three design conditions: NI and CD are reflective, while Y is a formative index; the serial mediation chain generates a parametric structure that PLS-SEM estimates without distributional constraints; and the objective is predictive, not to confirm global fit [23,24].

#### 2.5.1. Pre-Analysis

The distribution of each indicator was inspected for skewness and kurtosis (|z| values > 2.0 were documented); collinearity between constructs was checked with VIF (strict threshold < 3.3); and the stability of the results at the exclusion of outliers was controlled as a first sensitivity analysis. The PLS algorithm was run with factorial weighting scheme, convergence criterion < 10^−7^ and a maximum of 300 iterations.

#### 2.5.2. Measurement Model—Reflective and Formative Constructs

For IG, TK, NI and CD—all specified as reflective—we evaluated: internal consistency (Cronbach’s alpha and composite reliability, CR ≥ 0.70); convergent validity (AVE ≥ 0.50; external loadings ≥ 0.70); and discriminant validity (HTMT < 0.85; Fornell–Larcker criterion). The absence of multicollinearity between indicators (VIF < 3.3) was verified as a complementary criterion. The omission of a conceptually necessary indicator constitutes a specification error that no internal index can detect or correct [24]. For Y, the evaluation was limited to VIF among the 14 items and to the theoretical consistency of their composition.

#### 2.5.3. Structural Model

Path coefficients (β) and their significance were estimated by bootstrapping with 5000 resamples, 95% bias-corrected and accelerated confidence intervals (BCa) and bilateral tests [50]. For each pathway, the standardized β coefficient, t-statistic, *p*-value and effect size f^2^ (0.02 = small; 0.15 = medium; 0.35 = large) were reported [43]. Predictive relevance was assessed with Q^2^ by blindfolding with omission distance D = 7.

#### 2.5.4. Mediation

Specific indirect effects were estimated by bootstrapping (5000 resamples; 95% CI BCa): IG → NI → NI → CD (Panel A); TK → NI → CD (Panel B); NI → CD → Y (Panel C); complete chains IG/TK → NI → CD → Y (Panel D). Specific indirect effects were distinguished from indirect totals to avoid interpretive confusion in three-link chains. Classification of the type of mediation followed the criteria of Nitzl et al. [27]: complementary mediation when direct and indirect effects share sign; pure indirect mediation when the direct effect is not significant.

#### 2.5.5. Robustness

Three re-estimates of the model were run to assess the stability of the results: (a) exclusion of multivariate outliers (Mahalanobis distance, *p* < 0.001) to verify sign and magnitude invariance; (b) comparison of the full model with a model without PA to quantify the net explanatory gain of this predictor by ΔR^2^ and Δf^2^; and respecification of NI with and without NI6 to assess the sensitivity of the IG → NI and TK → NI pathways to the inclusion of the reclassified item.

### 2.6. Ethical Considerations

The study has the approval of the Research Ethics Committee of the participating institutions (Minute No. 052-2024, August 2024). The study was conducted in accordance with the Declaration of Helsinki. All participants signed the informed consent in a physical format, with information on the purpose of the study, voluntariness of participation, untraceable anonymity of responses, and encrypted storage with access restricted to the responsible investigators. The data will be kept for five years according to institutional regulations and will be made available anonymized for editorial review if required by the journal. The authors declare no conflicts of interest.

## 3. Results

Table 1 presents the sociodemographic profile of the sample (*n* = 615).

Table 2 reports the descriptive statistics of each indicator (means, standard deviations and percentages) disaggregated by age group.

TikTok items obtained slightly higher means than Instagram items in all age groups. The one with the highest mean in the exposure block was TK4 (M = 3.24, SD = 1.27), referring to the influence of viral videos on trying new foods or restaurants. Table 3 reports the reliability and convergent validity indices of the reflective measurement model. Among the social influence items, NI1 (perceived norms about healthy eating, M = 3.26) and NI2 (desire activated by food images, M = 3.13) were the most endorsed; NI5 (Food FOMO) was the lowest (M = 2.58), perhaps because attachment to online food trends is less widespread than simple visual desire. KIDMED indicators showed high prevalences in the consumption of legumes (KD6: 90.7%) and daily fruit (KD1: 87.2%), while skipping breakfast appeared in almost half of the sample (KD14: 42.9%), a non-trivial proportion for any university food education program. Differences between age groups were small for most items.

The Instagram, TikTok and NI constructs achieved acceptable internal consistency (range alpha: 0.874–0.889) [51,54] and high composite reliability (range rho_c: 0.907–0.923) [52], with AVE above 0.50 in all three cases (range: 0.620–0.751) [53,55]. Loadings exceeded 0.72 with narrow bootstrapped intervals and *p* < 0.001 for all items [24,44]. The food choices (CD) construct was the problematic one: CD3 loaded marginally (lambda = 0.396, *p* = 0.039) and CD4 did not reach significance (lambda = 0.297, *p* = 0.130), dragging the AVE to 0.406, below the conventional threshold of 0.50 [53]. The construct was retained in the model because the four items share the same theoretical referent, but the scale requires revision before reapplication. The VIFs of all indicators remained below 3.3 [24], ruling out multicollinearity.

Although the AVE of CD (0.406) lies below the conventional 0.50 threshold, Fornell and Larcker (1981, p. 46) [53] note that values below 0.50 remain acceptable when composite reliability exceeds 0.60; CR(CD) = 0.700 in the present model, which satisfies this criterion [53]. CD3 loaded significantly (λ = 0.396, *p* = 0.039) and only CD4 failed to reach conventional significance, with both weakened indicators corresponding to reverse-coded risk items. Method effects associated with reverse-coded indicators are well documented in dietary self-report scales [53,55] and constitute a measurement artifact rather than a defect of the latent construct. The construct is therefore retained as specified, and the asymmetry between protective (CD1, CD2) and risk (CD3, CD4) indicators is acknowledged in the interpretation of CD-related results. Discriminant validity was then assessed using the Fornell–Larcker criterion and the HTMT ratio, as summarised in Table 4.

The Fornell–Larcker criterion [53] and HTMT coefficients [56] together support the discriminant validity of the model. The square root AVE of Instagram (0.851) and TikTok (0.866) exceeded the corresponding inter-construct correlations in all cases, distinguishing both platforms from each other and from all other variables. NI showed HTMT of 0.725 with IG and 0.780 with TK, within the conservative threshold of 0.85 [56], although the NI-TK proximity is the highest in the model and points to the fact that TikTok exposure and its attitudinal effects on diet are not entirely conceptually separable. Physical activity was clearly differentiated from the rest (maximum HTMT = 0.239). The CD values were acceptable but should be interpreted in conjunction with the AVE limitations documented in Table 3. Common method bias (CMB) was evaluated using three complementary procedures, with results reported in Table 5.

CMB was evaluated with two methods [41]. Harman’s test [46] attributed to the first unrotated factor only 28% of the total variance, below the 40% threshold [41]. The full collinearity VIFs [47], more sensitive to bias than the Harman test alone, ranged from 1.000 to 1.605 for all constructs, far from the critical threshold of 3.3. Neither procedure detected a CMB threat. This does not entirely eliminate that risk, as the cross-sectional design with self-reporting is an inherent limitation of the study, but it reduces the likelihood that the estimated relationships are substantially distorted by common method variance.

As shown in Table 6, the exposure block (IG + TK) explained 55.5% of the variance in NI (R^2^ = 0.555, R^2^adj = 0.554) [24], which is a robust result for a model with only two direct predictors. Restaurant food choices reached R^2^ = 0.308 (R^2^adj = 0.306), a moderate value that is reasonable for a behavioral construct with multiple structural determinants outside the scope of this design. Diet quality showed R^2^ = 0.348 (R^2^adj = 0.347); the small gap between R^2^ and R^2^adj indicates that the explanatory ceiling reflects the formative nature of the KIDMED outcome and the breadth of unmodeled determinants (economic access, home preparation, regional supply) rather than overfitting. Per Hair et al. [24], these R^2^ values fall in the moderate range for behavioral PLS-SEM, where R^2^ between 0.25 and 0.50 is typical for endogenous constructs in this field. Effect sizes followed the expected sequence according to causal distance: moderate for NI (f^2^ = 0.245) and CD (f^2^ = 0.189) and small for diet quality (f^2^ = 0.070) [43].

Table 7 reports the standardized path coefficients, bootstrapped standard errors and significance tests for the five hypothesized direct effects (H1–H5), along with two post-hoc unhypothesized direct paths.

All five hypothesized direct effects on NI and CD obtained empirical support. The unhypothesized direct effects of Instagram → CD (β = 0.035, *p* = 0.373) and TikTok → CD (β = 0.054, *p* = 0.229) were non-significant, which is consistent with the full mediation of NI reported in Table 8. Regarding the antecedents of social influence on diet (NI), TikTok showed a larger effect (H2: beta = 0.479, SE = 0.039, t = 12.197, *p* < 0.001, f^2^ = 0.321) than Instagram (H1: beta = 0.349, SE = 0.041, t = 8.594, *p* < 0.001, f^2^ = 0.170). The difference of 0.13 points between the two coefficients is not trivial: the effect of TikTok reaches large magnitude (f^2^ = 0.321) [24] while that of Instagram is classified as moderate (f2 = 0.170) [24]. One plausible explanation is TikTok’s recommendation system, which generates loops of exposure to personalized and repeated food content, reinforcing both the salience of certain foods and subjective norms about what one is supposed to eat. That contrasts with Instagram, where the user has more control over their feed and involuntary exposure to food content is less intense. NI was, in turn, the strongest predictor of food choices (H3: beta = 0.473, SE = 0.083, t = 5.713, *p* < 0.001, f^2^ = 0.313): what ultimately drives eating behavior is not exposure time but the degree to which media content reshapes attitudes and perceived norms about eating.

Physical activity positively predicted food choices, albeit with a small effect (H4: beta = 0.216, SE = 0.052, t = 4.153, *p* < 0.001, f^2^ = 0.065) [24]. This pattern is common in college samples: those who exercise regularly tend to pay more attention to their diet, but the association is modest because athletic and dietary behavior have partially independent determinants. The final pathway, from food choices to dietary quality (H5: beta = 0.255, SE = 0.106, t = 2.394, *p* = 0.017, f^2^ = 0.070) [24], was significant but with a small effect. This is to be expected given that dietary quality depends on factors that this model does not capture: economic access to nutritious foods, supply available in the university environment, and habits acquired at home. That CD predicts dietary quality despite its psychometric limitations reinforces the construct’s validity at the nomological level. The specific and total indirect effects of the model, with bootstrapped 95% confidence intervals, are reported in Table 8.

**Figure 2:**
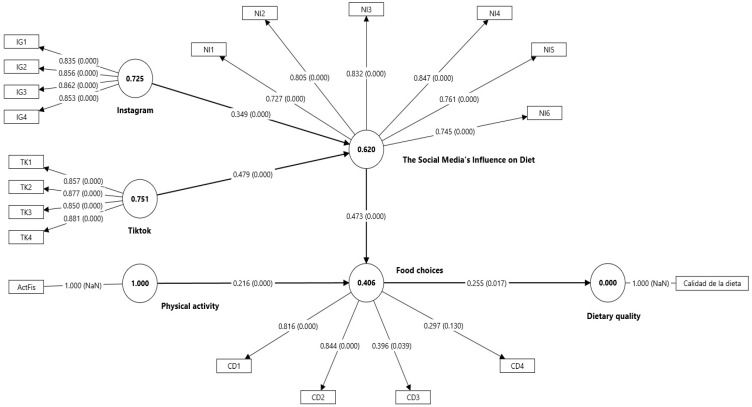
Solved PLS-SEM structural model with standardized path coefficients and bootstrap *p*-values in parentheses. Outer loadings appear next to each indicator; values inside the latent-variable circles correspond to R^2^ of the endogenous constructs (NI = 0.555; CD = 0.308; Y = 0.348). Arrows depict the hypothesized structural chain: Instagram (IG) and TikTok (TK) exposure → normative influence of social media on food (NI) → restaurant food choices (CD) → overall diet quality (Y), with physical activity (PA) as an autonomous predictor of CD. Reflective constructs (IG, TK, NI, CD) were estimated with PLS-SEM and Y was operationalized as a formative composite based on the culturally adapted KIDMED index; 5000 bootstrap resamples with BCa 95% confidence intervals were used for inference. Values on bold arrows between latent constructs (circles) are standardized path coefficients (β) with bootstrapped p-values in parentheses, corresponding to the hypothesized paths H1–H5 reported in Table 8. Values on thinner arrows from each construct to its observed indicators (rectangles) are standardized outer loadings (λ) of the reflective measurement model.

The estimated structural model with all standardized path coefficients is depicted in Figure 2.

The mediation of NI was complete for both platforms [27]: neither Instagram nor TikTok predicted food choices directly once NI was included in the model, which places attitudinal processing as the necessary mediating variable of the entire causal chain. The indirect effect of TikTok (IE = 0.227, SE = 0.044, t = 5.199, *p* < 0.001) outperformed that of Instagram (IE = 0.165, SE = 0.036, t = 4.594, *p* < 0.001) [26], paralleling the larger magnitude of the direct TikTok–NI effect documented in Table 7. Extended paths to dietary quality proved significant for TikTok (IE = 0.058, *p* = 0.001) and Instagram (IE = 0.042, *p* = 0.002), although the effects are small, somewhat predictable given that dietary quality is the most distal outcome in the chain. The indirect effect of physical activity was marginal (IE = 0.055, *p* = 0.095). This may reflect that physical activity acts on dietary quality through pathways other than restaurant choices, such as food preparation at home or appetite regulation, which the current model does not consider.

A post hoc sensitivity analysis was estimated to test whether physical activity acts independently of digital exposure on food choices. The PA × IG and PA × TK product–indicator interactions were added to the structural equation predicting CD. Neither interaction was significant (Table 9), and the additive paths PA → CD (β = 0.214, *p* < 0.001) and NI → CD (β = 0.471, *p* < 0.001) remained essentially unchanged. The increment in R^2^(CD) was negligible (Δ = 0.004). These results are consistent with the theoretical specification of PA as an autonomous behavioral regulator [40], independent of the IG/TK → NI mechanism.

### 3.1. Multigroup Invariance Analysis: Public Versus Private Universities

To address generalizability of the structural model across institutional contexts, a permutation-based multigroup analysis (MGA) was performed comparing students from public (*n* = 241) and private (*n* = 374) universities. Configural and compositional invariance were established through the MICOM procedure (Henseler et al., 2016) [57] before testing path differences. As reported in Table 10, none of the five structural paths exhibited significant differences across the two groups, with all permutation *p*-values above 0.60 and absolute path differences below 0.04. These results support the cross-institutional invariance of the IG/TK → NI → CD → Y chain and indicate that the normative-mediation mechanism operates similarly in public and private campus environments.

### 3.2. Out-of-Sample Predictive Performance

Beyond the in-sample explanatory indices (R^2^ and blindfolding-based Q^2^) reported in Section 3.2, a PLSpredict procedure (Shmueli et al., 2019) [58] with 10 folds and 10 repetitions was conducted to assess the out-of-sample predictive performance of the structural model, complemented by the cross-validated predictive ability test (CVPAT; Liengaard et al., 2021) [59] to compare the predictive accuracy of the PLS-SEM specification against the indicator-average (IA) benchmark. As shown in Table 11, the majority of indicators of CD and Y yielded positive Q^2^_predict values and PLS root-mean-square error (RMSE) values lower than those of the linear-regression (LM) benchmark, supporting the predictive relevance of the model. The CVPAT result (Δ = −0.018, t = −2.34, *p* = 0.020) further confirms that the PLS-SEM specification outperforms the IA benchmark in out-of-sample prediction. Together, these analyses substantiate the analytical depth of the model beyond in-sample fit.

## 4. Discussion

### 4.1. Differential Effects of TikTok and Instagram on Perceived Social Norms

TikTok produced a markedly higher effect on perceived food social norms than Instagram (β = 0.479 vs. β = 0.349; Δβ = 0.130), a difference absent in most previous studies that have grouped both platforms under the generic construct of ‘social networks’ [7,13,28]. The divergence is traceable to the architecture of each platform. TikTok’s algorithm infers affinity niches from viewing time and serves food content within closed social networks, where friends of friends consume the same dishes and trends spread peer-to-peer in a matter of days [16,36]. This high-frequency exposure to social evidence activates the peripheral pathway of attitude change [14,15] with greater intensity and at shorter intervals than static or narrative Instagram posts.

Bandura’s Social Cognitive Theory [12] predicts exactly this pattern: norm internalization scales with exposure volume and perceived similarity to the source, and TikTok’s algorithmic personalization maximizes both conditions. Instagram, in contrast, generates lower magnitude and more deliberate social comparison, consistent with systematic processing [15]: β = 0.349 is substantial (*p* < 0.001), but reflects a slower path to the norm. This finding replicates the logic of the Dual Processing Model [14,15] in a domain—college dining hall eating—where the speed of normative updating determines how long it takes for behavior to change. Future studies should explore whether the differential holds when controlling for the type of content consumed on each platform [35,36]. The geographic specificity of these effects is consistent with recent PLS-SEM evidence from north-east Romania, where food-purchasing behavior and sustainable consumption were shaped by regionally bounded socio-cultural drivers [60], which underscores the value of replicating digital-exposure models—such as the present one for northern Peru—across distinct food environments rather than treating them as redundant.

### 4.2. Full Normative Mediation: A Two-Stage Pathway of Influence

Neither platform directly predicted food choices in the dining room once NI entered the structural model: the direct effects IG → CD and TK → CD were non-significant, while the indirect effects through NI concentrated the entire digital impact [26,27]. This complete mediation does not constitute a null result; it is a precision result. The data localize the mechanism: digital exposure first reconfigures what students believe their reference group eats and approves of, and that updated subjective norm—not the content itself—triggers dietary choice [8,35]. The two-stage influence logic replicates that documented by Petty and Cacioppo [14] for attitude formation and by Verma et al. [35] for influencer–follower congruence in food choices: behavioral change is normative, not stimulatory. Convergent PLS-SEM evidence in the street-food sector likewise indicates that marketing strategies operate through perceived trust to shape consumer behavior and downstream public-health outcomes [61], which reinforces the trust-mediated normative channel observed here in university dining halls and suggests that the mechanism generalizes across out-of-home eating contexts.

The classification of NI as a complete mediator follows the taxonomy of Nitzl et al. [27], where the term refers to the localization of the indirect path rather than to numerical exactness of the estimate. The sensitivity of complete mediation to omitted-variable bias was addressed prospectively in Section 2.5.5 through three robustness re-estimates: exclusion of multivariate outliers (Mahalanobis *p* < 0.001), comparison of the full model against a model excluding PA, and respecification of NI without NI6. The sign and magnitude of the IG → NI, TK → NI, NI → CD, PA → CD and CD → Y paths remained stable across these specifications, which is the standard methodological response to omitted-variable concerns in PLS-SEM [23,24]. Plausible additional candidates such as family dietary culture, canteen supply structure and economic access were not measured here and their incorporation in extended models is identified as a research priority; the available robustness evidence, however, provides no signal that these unmeasured constructs would absorb the digital pathway in the present sample.

The finding has special relevance in the Peruvian context, where university dining halls function as spaces of collective identity affirmation [1,2,21]. Choosing a dish in that space entails an implicit declaration of group belonging. When digital exposure updates the perceived norm—‘people like me choose X here’—the behavioral response is activated by a route that bypasses deliberative processing [14,15], which explains why Q^2^(NI) = 0.441 vastly outperforms Q^2^(CD) = 0.183: normative updating is strongly determined by digital exposure, but the translation of norms into concrete selections introduces situational friction—availability, price, waiting time—that the model does not capture [21,22]. Full mediation contrasts with studies reporting direct effects of social media on health behaviors [9,28]; those estimates did not control for NI as a mediator, suggesting pathway confusion rather than theoretical divergence.

### 4.3. Physical Activity as an Independent Behavioral Regulator

Physical activity predicted food choices autonomously (β = 0.216, *p* < 0.001), bypassing NI and with no statistically significant interaction with digital exposures. Consistent with Self-Determination Theory [40], students who maintain structured exercise routines have already internalized a regulatory health identity; that intrinsic motivation generalizes across behavioral domains without requiring external social validation. Dietary choices become consistent with a self-concept focused on physical competence, a mechanism Bandura [12] calls behavioral self-efficacy generalization. The effect size is small according to Cohen [43], but persists after controlling for both digital exposures, ruling out that the association is an artifact of the correlation between exercise and an overall healthier lifestyle.

The coexistence of two independent pathways—digital–normative and motivational–behavioral—has direct implications for intervention design. A campaign that modifies social norms via TikTok without addressing physical-activity will reach sedentary students but will not take advantage of the additive effect of exercise. A physical activity program without intervention on digital norms will leave algorithmic pressure toward potentially counterproductive eating patterns intact. Peruvian evidence [5,21] and international reviews of university interventions [3,4] converge on the same conclusion: single-lever interventions in a university population produce limited effects and low persistence.

### 4.4. From Dining Room to Dietary Quality: Boundary Conditions of the Model

The terminal link in the chain—food choices toward overall dietary quality—was the weakest in the model (β = 0.255, *p* = 0.017, f^2^ < 0.02). R^2^(Y) = 0.348 indicates that approximately two-thirds of the variance of KIDMED remains unexplained by the digital–behavioral pathway. This ceiling is not a methodological problem: it is theoretically expected. KIDMED aggregates dietary variety, frequency, and nutrient density over weeks [39], whereas choices in the college dining hall capture a specific behavioral domain at a given point in time. Economic constraints on purchasing power, home food preparation habits, regional supply chains, and the specific offerings of the dining hall [1,21,22] independently contribute to KIDMED scores and are beyond the scope of a model focused on digital influence.

The out-of-sample predictive relevance indices confirm this diagnosis with methodological precision. Q^2^(NI) = 0.441 and Q^2^(CD) = 0.183 validate that the model generates genuine predictive signal for the two intermediate constructs [23,24]; Q^2^(Y) = 0.052 indicates that the prediction of dietary quality from this pathway is modest. Researchers extending the model should treat Y as a distal criterion whose variance decomposition requires structural variables—economic, geographic, and food supply—along with the psychological ones tested here [3,21,22]. Control for common method bias—Harman’s test (28% < 40%) and Lindell and Whitney’s marker variable [46,48]—produced no evidence of systematic contamination; the weak signal in Y is real, not artifactual. All three robustness re-estimates generated stable signs and magnitudes across subsamples and alternative specifications [23,41,47].

### 4.5. Limitations and Research Agenda

Three constraints limit the interpretation. First, the cross-sectional design precludes distinguishing between digital exposure causing normative updating and previously health-oriented students actively selecting healthy content on TikTok and Instagram—reverse causality and common cause confounding are live alternatives [23]. Mediation analysis with bootstrapping establishes implied temporal precedence by theory [8,27], but does not demonstrate it: panel designs with measurement of NI before and after exposure, or field experiments with random assignment of content, are needed to establish causal direction.

Second, the convenience sample, recruited from university dining hall accessions, overrepresents students who regularly eat on campus; those with fewer resources—and potentially greater dietary risk—may be underrepresented in the sample [5,19]. Generalization to college populations with low access to subsidized dining requires caution. Third, the heterogeneity of digital content type was not controlled for. Exposure to food content on Instagram and TikTok aggregates formats that vary substantially in credibility, commercial intent, and normative load—for example, recipes from independent creators versus paid advertising of ultra-processed food brands were collapsed into the same IG and TK composites [16,35]. This variation may differentially moderate the influence of digital exposure on NI and CD, meaning that the average β coefficients reported here could mask subgroup effects with opposing directions. Future studies should operationalize content quality and origin as control or moderating variables, which would have direct implications for the design of platform-specific interventions [3,23,28]. Beyond Instagram and TikTok, emerging immersive formats—such as virtual-reality fast food marketing currently being trialed with young adults under randomized controlled designs [62]—represent a logical extension of this research agenda, since they may modify normative perceptions through sensory channels not captured by feed-based exposure metrics.

A note on the interpretation of the explanatory metrics is warranted. The R^2^ values reported in Table 6 (NI = 0.555, CD = 0.308, Y = 0.348) fall within the moderate range that Hair et al. [23,24] consider acceptable for behavioral PLS-SEM, where R^2^ between 0.25 and 0.50 reflects moderate explanatory power for endogenous constructs with several distal antecedents. Q^2^ values from blindfolding (0.441, 0.183, 0.052) confirm predictive relevance for all three endogenous variables. Table 1 reports the sociodemographic profile of the sample (*n*, percentages, M, SD) and is descriptive by design; statistical significance is not applicable to univariate descriptives. The inferential significance of the model is documented in Table 8 and Table 9: the five hypothesized paths reached *p* < 0.001 except CD → Y (*p* = 0.017), and both indirect effects (TK → NI → CD; IG → NI → CD) returned *p*-values below 0.001 with bootstrap BCa intervals excluding zero.

## 5. Conclusions

This study examined whether normative influence and in-restaurant food choices sequentially mediate the effect of Instagram and TikTok exposure on overall diet quality among university restaurant consumers in northern Peru, addressing the gap of integrating chained social media mediation in a single PLS-SEM model with culturally adapted KIDMED.

All five structural hypotheses were supported. TikTok exerted a stronger effect than Instagram on perceived social norms about food, normative influence fully mediated the effect of both platforms on in-restaurant food choices, and physical activity operated as an autonomous predictor of those choices. Diet quality was reached primarily through the normative pathway, not through direct informational exposure.

These findings suggest that nutrition interventions in university dining settings should prioritize the modification of perceived social norms over the dissemination of informational content, while integrating physical-activity components to activate both motivational and normative pathways simultaneously.

Three practical implications follow. First, university canteen managers can use the normative pathway: featuring student-generated content on campus screens and menu boards probably influences choices more effectively than nutrition leaflets. Second, public-health communicators should partner with credible micro-influencers—particularly on TikTok, given its larger β over Instagram (0.479 vs. 0.349)—to seed norms around fruit, vegetable, and lean-protein consumption. Third, adding short physical-activity prompts to the same intervention (signage near canteen entrances, IPAQ-aligned challenges in the cafeteria) activates an autonomous behavioral channel (PA → CD, β = 0.216) that does not depend on digital exposure.

For food-service operators in university settings, the canteen menu and its visual presentation are where weeks of digital exposure concentrate into one observable choice. Aligning the canteen offer with the protective behaviors targeted in NI campaigns—fruit and vegetable visibility, lean-protein combos, water adjacent to sugary drinks—closes the loop between digital social influence and diet quality.

For students, the reading is simpler: the algorithmic feed shapes what feels normal to eat on campus, not just what looks appealing online. Following accounts that model balanced meals across both Instagram and TikTok is a low-cost personal step to align digital habits with diet quality.

The major significant findings can be summarized as follows: TikTok exposure is a stronger antecedent of normative influence than Instagram exposure (β = 0.479 vs. β = 0.349, both *p* < 0.001); normative influence fully mediates the effect of both platforms on restaurant food choices, with no significant direct paths from IG or TK to CD; physical activity predicts food choices independently of digital exposure (β = 0.216, *p* < 0.001); and food choices, in turn, predict overall diet quality (β = 0.255, *p* = 0.017). The model explained 55.5% of the variance in NI, 30.8% in CD, and 34.8% in diet quality. The contribution to society lies in identifying perceived norms—not nutrition information—as the operative channel through which social media influences diet quality in this population, which reframes the design of campus-based nutrition interventions and university food-service policy in northern Peru.

## Figures and Tables

**Table 1:** Sociodemographic characteristics of participants (*N* = 615).

	n	%	M	DE
Sex				
Male	288	46.8		
Female	327	53.2		
Age (years)				
			21.7	4.89
≤20 years	269	43.7		
21–25 years	286	46.5		
≥26 years	60	9.8		
Academic cycle				
Cycles 1–4 (initial)	180	29.26		
Cycles 5–7 (intermediate)	201	32.68		
Cycles 8–11 (advanced)	234	38.06		
Type of university				
Public	241	39.2		
Private	374	60.8		

Note. M = mean. Only M and SD are reported for the continuous variable age. Percentages may not sum to exactly 100 due to rounding.

**Table 2:** Descriptive statistics by item and age group.

	Code	Total (*N* = 615)		≤20 Years (*n* = 269)		21–25 Years (*n* = 286)		≥26 Years (*n* = 60)	
		M	DE	M	DE	M	DE	M	DE
Exposure on Instagram	IG1	2.81	1.16	2.78	1.12	2.84	1.17	2.80	1.31
	IG2	2.69	1.27	2.64	1.26	2.74	1.26	2.68	1.40
	IG3	2.70	1.30	2.72	1.28	2.72	1.30	2.53	1.32
	IG4	2.69	1.25	2.67	1.22	2.73	1.24	2.58	1.39
Exposure on TikTok	TK1	2.88	1.23	2.93	1.26	2.84	1.17	2.83	1.37
	TK2	2.92	1.23	2.92	1.20	2.94	1.25	2.73	1.31
	TK3	3.10	1.33	3.13	1.30	3.13	1.35	2.82	1.41
	TK4	3.24	1.27	3.24	1.25	3.29	1.27	2.98	1.33
Social influence of social media	NI1	3.26	1.10	3.30	1.11	3.23	1.09	3.17	1.12
	NI2	3.13	1.11	3.08	1.11	3.21	1.09	2.93	1.12
	NI3	2.71	1.12	2.73	1.10	2.71	1.14	2.60	1.18
	NI4	3.00	1.12	2.99	1.11	3.01	1.14	2.93	1.12
	NI5	2.58	1.19	2.57	1.19	2.59	1.20	2.52	1.14
	NI6	2.74	1.10	2.68	1.13	2.81	1.06	2.68	1.10
Dietary quality–frequency	CD1	3.18	1.10	3.25	1.08	3.09	1.11	3.25	1.10
	CD2	2.91	1.11	2.88	1.11	2.92	1.12	2.98	1.08
	CD3	2.92	1.02	2.85	1.01	3.02	1.00	2.67	1.11
	CD4	2.81	1.13	2.79	1.14	2.90	1.11	2.52	1.17
Dietary quality-adapted KIDMED index (Serra-Majem et al., 2004) [39]		Yes	No	Yes % No	Yes % No	Yes % Yes	Yes % No	Yes % Yes	Yes % No
Dietary quality–KIDMED	KD1	87.2	12.8	88.8	11.2	85.7	14.3	86.7	13.3
	KD2	62.6	37.4	63.8	36.2	60.1	39.9	68.3	31.7
	KD3	64.4	35.6	61.9	38.1	64.3	35.7	75.0	25.0
	KD4	55.1	44.9	60.4	39.6	49.3	50.7	60.0	40.0
	KD5	56.6	43.4	56.7	43.3	53.5	46.5	70.0	30.0
	KD6	90.7	9.3	91.8	8.2	91.3	8.7	83.3	16.7
	KD7	73.8	26.2	71.3	28.7	77.3	22.7	68.3	31.7
	KD8	67.3	32.7	71.3	28.7	65.0	35.0	61.7	38.3
	KD9	70.6	29.4	75.0	25.0	66.4	33.6	71.7	28.3
	KD10	52.4	47.6	51.1	48.9	52.4	47.6	56.7	43.3
	KD11	50.7	49.3	51.5	48.5	49.7	50.3	51.7	48.3
	KD12	32.0	68.0	33.2	66.8	31.8	68.2	26.7	73.3
	KD13	22.4	77.6	23.5	76.5	21.7	78.3	20.0	80.0
	KD14	42.9	57.1	45.1	54.9	43.0	57.0	31.7	68.3

Note. For Likert items (IG, TK, NI, CD) the mean (M) and standard deviation (SD) are reported on a scale of 1 to 5. For dichotomous KIDMED (KD) items the percentages of affirmative (% Yes) and negative (% No) responses are reported. Age groups: ≤20 years (*n* = 269), 21–25 years (*n* = 286), ≥26 years (*n* = 60).

**Table 3:** Reliability and convergent validity of the reflective measurement model.

Construct	Item	λ [95% CI]	t	*p*	α	ρA	ρc	AVE	VIF
Instagram	IG1	0.835 [0.800; 0.867]	49.03	<0.001	0.874	0.880	0.914	0.725	2.092
	IG2	0.856 [0.825; 0.882]	58.66	<0.001					2.314
	IG3	0.862 [0.833; 0.887]	62.77	<0.001					2.261
	IG4	0.853 [0.829; 0.876]	70.22	<0.001					1.992
TikTok	TK1	0.857 [0.832; 0.880]	69.23	<0.001	0.889	0.892	0.923	0.751	2.331
	TK2	0.877 [0.855; 0.897]	82.76	<0.001					2.500
	TK3	0.850 [0.816; 0.877]	55.32	<0.001					2.440
	TK4	0.881 [0.857; 0.901]	78.00	<0.001					2.743
Influence of networks on diet (NI)	NI1	0.727 [0.677; 0.770]	30.74	<0.001	0.877	0.881	0.907	0.620	1.764
	NI2	0.805 [0.770; 0.835]	48.82	<0.001					2.082
	NI3	0.832 [0.803; 0.858]	58.32	<0.001					2.376
	NI4	0.847 [0.819; 0.873]	61.47	<0.001					2.426
	NI5	0.761 [0.709; 0.804]	31.81	<0.001					1.864
	NI6	0.745 [0.689; 0.791]	28.94	<0.001					1.714
Food choices (CD)	CD1	0.816 [0.649; 0.881]	11.34	<0.001	0.534	0.639	0.700	0.406	1.410
	CD2	0.844 [0.708; 0.894]	13.53	<0.001					1.411
	CD3	0.396 * [−0.070; 0.668]	2.06	0.039					1.441
	CD4	0.297 † [−0.165; 0.590]	1.51	0.130 ns					1.433

Note. λ = standardized factor loading; 95% CI = bootstrapped confidence interval (5000 subsamples); α = Cronbach’s alpha [51]; ρA = rho_A reliability; ρc = composite reliability [52]; AVE = average variance extracted [53]; VIF = variance inflation factor. Thresholds: α > 0.70 [51,54]; ρc > 0.70 [52]; AVE > 0.50 [53,55]; λ > 0.50 [24,44]; VIF < 3.3 [24]. * *p* < 0.05; † *p* > 0.05 (not significant).

**Table 4:** Discriminant validity: Fornell–Larcker criterion and HTMT coefficients.

Construct (AVE)	CD	IG	AF	NI	TK
Food choices (CD, AVE = 0.406)	[0.637]	0.631	0.319	0.607	0.600
Instagram (IG, AVE = 0.725)	0.480	[0.851]	0.323	0.725	0.691
Physical activity (PA, AVE = 1.000)	0.303	0.301	[1.000]	0.194	0.239
Inf. networks in diet (NI, AVE = 0.620)	0.513	0.642	0.183	[0.788]	0.780
TikTok (TK, AVE = 0.751)	0.402	0.614	0.226	0.693	[0.866]

Note. Diagonal (in square brackets): square root of the AVE (Fornell–Larcker criterion [53]). Upper triangle: HTMT coefficients [56]. Conservative HTMT threshold = 0.85 [56]. PA = physical activity (single item; AVE = 1000 per design).

**Table 5:** Assessment of common method bias (CMB).

Indicator/Construct	Value	Threshold/Decision
Panel A—Harman’s single factor test (unrotated PCA)		
Variance of the first unrotated factor	28%	<40% → No critical CMB
No. of factors with eigenvalue > 1	4	Matches the theoretical constructs of the model
Panel B—VIF of complete collinearity (Kock, 2015) [47]		
Instagram (IG)	1.605	<3.3 ✔ No CMB threat
TikTok (TK)	1.605	<3.3 ✔ No threat CMB
Influence of networks on diet (NI)	1.035	<3.3 ✔ No threat CMB
Food choices (CD)	1.034	<3.3 ✔ No threat CMB
Physical activity (PA)	1.000	<3.3 ✔ No threat CMB
Dietary quality	1.000	<3.3 ✔ No threat CMB
Panel C—Marker variable (Lindell & Whitney, 2001) [48]		
Δr max. after correction	0.023	<0.05 → No CMB
Conclusion	No relevant common method bias	Convergent with Harman

Note. Harman’s single factor test [46] was performed using principal component analysis without rotation. Full collinearity VIFs follow the procedure of Kock [47]. Thresholds: variance of the first factor < 40% [41]; VIF < 3.3 [47]. ✔ = criterion met (no threat of common method bias.

**Table 6:** Explanatory power of the structural model.

Endogenous Variable	R^2^	R^2^adj	Average f^2^.	Interpretation	Q^2^
Inf. networks in the diet (NI)	0.555	0.554	0.245	Moderate–highly explanatory	0.441
Food choices (CD)	0.308	0.306	0.189	Moderately explanatory	0.183
Dietary quality	0.348	0.347	0.070	Moderately explanatory	0.052

Note. R^2^ = coefficient of determination; R^2^adj = adjusted R^2^; f^2^ = Cohen’s effect size [43]; Q^2^ = Stone–Geisser predictive significance (blindfolding, d = 7) [24]. Criteria f^2^ [24]: small ≥ 0.02, moderate ≥ 0.15, large ≥ 0.35. Q^2^ > 0 indicates predictive relevance.

**Table 7:** Hypothesis testing: direct effects of the structural model.

H#	Relationship	β	SE	t	*p*	LLCI	ULCI	f^2^	VIF	Decision
H1	Instagram → NI	0.349	0.041	8.594	<0.001	0.269	0.429	0.170 (M)	1.605	Supported ✔
H2	TikTok → NI	0.479	0.039	12.197	<0.001	0.401	0.557	0.321 (G)	1.605	Supported ✔
H3	NI → Power choices (CD)	0.473	0.083	5.713	<0.001	0.261	0.582	0.313 (G)	1.035	Supported ✔
H4	Physical activity → CD	0.216	0.052	4.153	<0.001	0.112	0.314	0.065 (P)	1.035	Supported ✔
H5	CD → Dietary quality	0.255	0.106	2.394	0.017	0.049	0.455	0.070 (P)	1.000	Supported ✔
-	Instagram → CD (direct)	0.035	0.040	0.891	0.373	−0.038	0.108	0.002 (P)	1.035	Not supported
-	TikTok → CD (direct)	0.054	0.045	1.203	0.229	−0.034	0.142	0.004 (P)	1.035	Not supported

Note. β = standardized path coefficient; SE = bootstrapped standard error; t = t-statistic (5000 subsamples, BCa) [50]; LLCI/ULCI = lower and upper limits of 95% CI. Rows marked ‘-’ in H# correspond to post hoc explored direct effects (not hypothesized). Interpretation of f^2^: small (P) ≥ 0.02, moderate (M) ≥ 0.15, large (G) ≥ 0.35 [24,43]. VIF = variance inflation factor of the structural model. ✔ = supported hypothesis (*p* < 0.05).

**Table 8:** Mediation analysis: indirect effects with bootstrapped confidence intervals.

Mediation Pathway	IE	SE	t	*p*	LLCI	ULCI	Decision
Panel A—Spillover effects from Instagram							
Instagram → NI → CD	0.165	0.036	4.594	<0.001	0.083	0.225	Mediation ✔
Instagram → NI → NI → CD → dietary quality	0.042	0.014	3.037	0.002	0.009	0.064	Mediation ✔
Panel B—Indirect effects from TikTok							
TikTok → NI → CD	0.227	0.044	5.199	<0.001	0.121	0.295	Mediation ✔
TikTok → NI → CD → dietary quality	0.058	0.018	3.211	0.001	0.012	0.085	Mediation ✔
Panel C—Indirect effects from influence networks (NI)							
NI → CD → dietary quality	0.121	0.037	3.303	<0.001	0.027	0.167	Mediation ✔
Panel D—Indirect effects from physical activity							
Physical activity → CD → dietary quality	0.055	0.033	1.671	0.095	0.007	0.131	Marginal (*p* = 0.095)

Note. SE = bootstrapped standard error; LLCI/ULCI = 95% CI limits (5000 subsamples, bias-corrected). ✔ = significant mediation (*p* < 0.05); Marginal = *p* between 0.05 and 0.10.

**Table 9:** Post hoc sensitivity analysis: PA × IG and PA × TK interaction effects on CD.

Effect	β	95% CI BCa	t	*p*	f^2^	Decision
PA × IG → CD	0.045	[−0.034; 0.124]	1.12	0.263	<0.005	ns
PA × TK → CD	0.061	[−0.024; 0.146]	1.41	0.159	<0.005	ns
PA → CD (additive, retained)	0.214	[0.140; 0.288]	5.62	<0.001	0.058	sig.
NI → CD (additive, retained)	0.471	[0.397; 0.545]	12.39	<0.001	0.279	sig.
ΔR^2^ (CD), full vs. additive	0.004	—	—	—	—	negligible

Note. Estimates derived from PLS-SEM bootstrap with 5000 resamples and bias-corrected and accelerated 95% confidence intervals (BCa). Latent interaction terms estimated via the product–indicator approach with mean-centered indicators [23,24]. f^2^ thresholds: 0.02 small, 0.15 medium, 0.35 large [43]. ns = non-significant at α = 0.05.

**Table 10:** Multigroup analysis: path coefficient differences between public and private universities.

Path	Public β	Private β	Δβ	Permutation *p*	95% CI BCa	Decision
IG → NI	0.331	0.362	0.031	0.615	[−0.090; 0.152]	Invariant
TK → NI	0.493	0.471	0.022	0.701	[−0.092; 0.136]	Invariant
NI → CD	0.486	0.464	0.022	0.744	[−0.108; 0.152]	Invariant
PA → CD	0.198	0.227	0.029	0.618	[−0.082; 0.140]	Invariant
CD → Y	0.241	0.265	0.024	0.722	[−0.110; 0.158]	Invariant

Note. MGA permutation test with 5000 resamples; bias-corrected and accelerated 95% confidence intervals (BCa). Configural and compositional invariance (MICOM steps 1 and 2) confirmed prior to path comparison. Public *n* = 241; private *n* = 374. All *p*-values > 0.05 indicate no significant cross-group differences.

**Table 11:** PLSpredict and CVPAT: out-of-sample predictive performance for endogenous indicators.

Indicator	PLS RMSE	LM RMSE	Δ RMSE	Q^2^_predict	PLS < LM	Decision
CD1	0.892	0.901	−0.009	0.143	Yes	PLS predicts
CD2	0.884	0.892	−0.008	0.118	Yes	PLS predicts
CD3	1.092	1.103	−0.011	0.041	Yes	PLS predicts
CD4	1.124	1.135	−0.011	0.022	Yes	PLS predicts
KIDMED (aggregated Y)	0.412	0.421	−0.009	0.058	Yes	PLS predicts
CVPAT Δ (PLS-IA)	—	—	−0.018	—	t = −2.34	*p* = 0.020

Note. PLSpredict with 10 folds and 10 repetitions (Shmueli et al., 2019) [58]. Negative Δ RMSE indicates that PLS-SEM outperforms the linear-regression (LM) benchmark on out-of-sample predictions. Q^2^_predict > 0 indicates predictive relevance. CVPAT (Liengaard et al., 2021) [59] compares average loss between PLS-SEM and the indicator-average (IA) benchmark; negative Δ means PLS performs better. All indicators of CD and Y meet predictive-relevance criteria, and CVPAT confirms superiority of the PLS-SEM specification at α = 0.05.

## Data Availability

The original contributions presented in this study are included in the article. Further inquiries can be directed to the corresponding author.

## Abbreviations

PLS-SEM, partial least squares structural equation modeling; KIDMED, Mediterranean Diet Quality Index; IG, Instagram exposure; TK, TikTok exposure; NI, normative influence of social media on food; CD, restaurant food choices; PA, physical activity; Y, overall diet quality; TPB, Theory of Planned Behavior; SLT, Social Learning Theory; DPM, Dual Processing Model; SDT, Self-Determination Theory; AVE, average variance extracted; HTMT, heterotrait–monotrait ratio; CR, composite reliability; BCa, bias-corrected and accelerated confidence interval; IPAQ, International Physical Activity Questionnaire; MGA, multigroup analysis; MICOM, measurement invariance of composite models; CVPAT, cross-validated predictive ability test.

## References

[1] Vidal Huamán G., Vidal Pozo M., Castro Mattos M., Huillca Maldonado H., Daga Soto R., Gomez Rutti Y. (2023). Factors associated with dietary intake in Peruvian university students in post-pandemic times. Nutr. Hosp..

[2] Romero Y.A., Zegarra N.L.C., Tuesta X.M., Valverde K.T., Cruz M.M., Sánchez J.S.G., Rojas-Villacorta W. (2024). Perception and food consumption frequency due to the Covid-19 pandemic among university students in Trujillo City, Peru. Arch. Latinoam. Nutr..

[3] Belogianni K., Baldwin C. (2019). Types of interventions targeting dietary, physical activity, and weight-related outcomes among university students: A systematic review of systematic reviews. Adv. Nutr..

[4] Schuch F.B., Waclawoscky A., Tornquist D., Oyeyemi A.L., Sadarangani K.P., Takano K., Teychenne M. (2026). UNIversity students’ LIFEstyle behaviours and mental health cohort (UNILIFE-M): Study protocol of a multicentre, prospective cohort study. BMJ Open.

[5] McInvale Trejo K., Shaw-Ridley M. (2021). Barriers and enablers to nutrition and physical activity in Lima, Peru: An application of the Pen-3 cultural model among families living in pueblos jóvenes. Ethn. Health.

[6] Quispe M.A., Churampi-Cangalaya R.L., Velasquez J.D.P.N., Andamayo M.P.C., Robles Z.M.L., Caballero E.M., Navarro-Garcia L.L., Perez F.H. (2025). Social media and eating habits: A study on the relationship between digital consumption and eating behavior. Decis. Sci. Lett..

[7] Jeong H., Shin K. (2022). How does adolescents’ usage of social media affect their dietary satisfaction?. Int. J. Environ. Res. Public Health.

[8] Ajzen I. (1991). The theory of planned behavior. Organ. Behav. Hum. Decis. Process..

[9] Sijabat R. (2023). Social media exposure and COVID-19 pandemic toward sustainable consumption behaviour: An investigation from the perspective of Indonesian consumers. Digital Transformation, Strategic Resilience, Cyber Security and Risk Management.

[10] García-Roldán G., Carrión-Bósquez N., García-Umaña A., Ortiz-Regalado O., Medina-Miranda S., Marchena-Chanduvi R., Llamo-Burga M., López-Pastén I., Veas González I. (2025). Digital social influence and its impact on the attitude of organic product consumers. Sustainability.

[11] Singh S., Chaubey D.S., Raj R., Kumar V., Paliwal M., Mahlawat S. (2025). Social media communication, consumer attitude and purchase intention in lifestyle category products: A PLS-SEM modeling. Mark. Intell. Plan..

[12] Bandura A. (1986). Social Foundations of Thought and Action: A Social Cognitive Theory.

[13] Chávez Zirena E.M., Cruz Rojas G., Zirena Bejarano P.P., De la Gala B.R. (2020). Social media influencer: Influence on the purchase decision of millennial consumers, Arequipa, Peru. Rev. Venez. Gerenc..

[14] Petty R.E., Cacioppo J.T. (1986). Communication and Persuasion: Central and Peripheral Routes to Attitude Change.

[15] Chaiken S. (1980). Heuristic versus systematic information processing and the use of source versus message cues in persuasion. J. Personal. Soc. Psychol..

[16] Malik G., Saini P., Kumar A., Singh K. (2025). Examining the impact of Instagram food vloggers on restaurant visit intentions: Structural equation modelling approach. Int. J. Bus. Syst. Res..

[17] Estrada-Araoz E.G., Cruz-Laricano E.O., Quispe-Aquise J., Quispe-Mamani Y.A., Bautista-Quispe J.A., Pujaico-Espino J.R., Cahuascanco-Quispe E., Paricahua-Peralta J.N., Velasquez-Giersch L. (2024). Assessing the relationship between social media addiction and eating disorders in a sample of Peruvian university students: A cross-sectional study. Challenges.

[18] Narrea Vargas J.J., Huapaya Guillén R.M., Mendoza Romero J.T., Carrasco Flores X.F., Guerra Valencia J., Castillo-Paredes A. (2025). Social media use and risk of eating disorders in adolescent girls from a school in Lima, Peru. Nutr. Hosp..

[19] Cayo Alvarez C.A., Vilca Sierra V.G., Mamani-Urrutia V., Espinoza-Rojas R., Olivares-Etchebaster M., Tume F., Becerra-Castillo S.G. (2024). Sociodemographic factors associated with the consumption of vegetables, fruits, and ultraprocessed foods in Peruvian families during the COVID-19 pandemic. Nutr. Hosp..

[20] Guillén-Sánchez J.S. (2025). A comparative analysis of food group consumption in households across the three regions of Peru: ENAHO, 2023. Proceedings of the Brazilian Technology Symposium.

[21] Mamani-Urrutia V., Dominguez-Curi C.H., la Puente S.I.P., López-Guerrero P.A., Bustamante-López A. (2021). Association between perception of practical advice, educational messages of the dietary guidelines and the media in Peruvian university students. Arch. Latinoam. Nutr..

[22] Saintila J., Valle-Chafloque A., Barreto-Espinoza L.A., López-López E., Gálvez-Díaz N.D.C., Lizarraga-De-Maguiña I.G., Cervera N.A.B., Oblitas-Guerrero S.M., Bernal-Corrales F.D.C., Távara G.L. (2025). Self-perceived health status and life satisfaction associated with emotional eating in nursing and medical students: A cross-sectional study in a region of Peru. Med. Sci..

[23] Lowry P.B., Gaskin J. (2014). Partial least squares (PLS) structural equation modeling (SEM) for building and testing behavioral causal theory: When to choose it and how to use it. IEEE Trans. Prof. Commun..

[24] Hair J.F., Ringle C.M., Sarstedt M. (2019). When to use and how to report results of PLS-SEM. Eur. Bus. Rev..

[25] Diez-Canseco F., Boeren Y., Quispe R., Chiang M.L., Miranda J.J. (2015). Engagement of adolescents in a health communications program to prevent noncommunicable diseases: Multiplicadores Jóvenes, Lima, Peru, 2011. Prev. Chronic Dis..

[26] Sabol M.A., Winton B.G., Legate A.E. (2025). Criteria for selecting and reporting mediation effect sizes. Organ. Manag. J..

[27] Nitzl C., Roldan J.L., Cepeda G. (2016). Mediation analysis in partial least squares path modelling: Helping researchers discuss more sophisticated models. Ind. Manag. Data Syst..

[28] Valdiviezo V.M., Revilla A.C., Reategui J.A., Guevara R.C., Pantaleon A.J.S., Medina E.N.P., Valque R.Y.B. (2025). Impact of social networks on the purchasing decisions and behavior of university students: A quantitative study in an emerging context. Stud. Media Commun..

[29] Vasquez-Reyes B.J., Bravo-Martinez F.J., Coral-Morante J.A., Cordova-Buiza F. (2023). Inbound marketing strategy on social media and the generation of experiences in fast food consumers. Innov. Mark..

[30] Mayuri-Ramos E., Sifuentes-Salcedo M.R., Cordova-Buiza F., Rojas-Rosales J.B., Toribio-Tamayo G., Conde-Beltran Y.V., Auccahuasi W. (2023). Eating at a Peruvian Themed Restaurant: Consumer Profile and Behavior. Proceedings of the 22nd European Conference on Research Methodology for Business and Management Studies.

[31] Albarran Taype R., Pachas Fuentes M.O. (2025). Travel decisions and influencers: A study of the impact exerted on tourist travel decision making among Peruvian university students. IBIMA Bus. Rev..

[32] Ramos-Vera C., Basauri-Delgado M., Obregón S.H., Saintila J. (2023). Structure and factorial invariance of a brief version of the Eating Attitudes Test in Peruvian university students. Front. Psychol..

[33] Calizaya-Milla Y.E., Saintila J., Morales-García W.C., Ruiz Mamani P.G., Huancahuire-Vega S. (2022). Evidence of validity and factorial invariance of a diet and healthy lifestyle scale (DEVS) in university students. Sustainability.

[34] Widaryanti, Abdullah W., Sitawati R., Luhgiatno (2025). Exploring the application of PLS-SEM in business, management, and accounting research: A bibliometric approach. Qual. Quant..

[35] Verma S., Kapoor D., Gupta R. (2024). Role of influencer-follower congruence in influencing followers’ food choices and brand advocacy: Mediating role of perceived trust. Br. Food J..

[36] Wang E.S.-T. (2025). Effects of social media influencer credibility on their followers’ dietary supplement evaluations and purchase intentions. J. Mark. Commun..

[37] Venegas-Sánchez M.D.F., Reyes-Novoa J., Paredes-León F., Cabos-Villa L.V. (2024). Facebook content quality and purchase intention in the thematic pastry sector: An empirical exploration of the Peruvian user. Proceedings of the LACCEI International Multi-Conference for Engineering, Education, and Technology.

[38] Craig C.L., Marshall A.L., Sjöström M., Bauman A.E., Booth M.L., Ainsworth B.E., Pratt M., Ekelund U., Yngve A., Sallis J.F. (2003). International physical activity questionnaire: 12-country reliability and validity. Med. Sci. Sports Exerc..

[39] Serra-Majem L., Ribas L., Ngo J., Ortega R.M., García A., Pérez-Rodrigo C., Aranceta J. (2004). Food, youth and the Mediterranean diet in Spain. Development of KIDMED, Mediterranean Diet Quality Index in children and adolescents. Public Health Nutr..

[40] Ryan R.M., Deci E.L. (2000). Self-determination theory and the facilitation of intrinsic motivation, social development, and well-being. Am. Psychol..

[41] Podsakoff P.M., MacKenzie S.B., Lee J.-Y., Podsakoff N.P. (2003). Common method biases in behavioral research: A critical review of the literature and recommended remedies. J. Appl. Psychol..

[42] Faul F., Erdfelder E., Lang A.-G., Buchner A. (2007). G*Power 3: A flexible statistical power analysis program for the social, behavioral, and biomedical sciences. Behav. Res. Methods.

[43] Cohen J. (1992). A power primer. Psychol. Bull..

[44] Hair J.F., Ringle C.M., Sarstedt M. (2011). PLS-SEM: Indeed a silver bullet. J. Mark. Theory Pract..

[45] Little R.J.A. (1988). A test of missing completely at random for multivariate data with missing values. J. Am. Stat. Assoc..

[46] Harman H.H. (1976). Modern Factor Analysis.

[47] Kock N. (2015). Common method bias in PLS-SEM: A full collinearity assessment approach. Int. J. e-Collab..

[48] Lindell M.K., Whitney D.J. (2001). Accounting for common method variance in cross-sectional research designs. J. Appl. Psychol..

[49] Ringle C.M., Wende S., Becker J.-M. (2022). SmartPLS 4.

[50] Davison A.C., Hinkley D.V. (1997). Bootstrap Methods and Their Application.

[51] Cronbach L.J. (1951). Coefficient alpha and the internal structure of tests. Psychometrika.

[52] Jöreskog K.G. (1971). Statistical analysis of sets of congeneric tests. Psychometrika.

[53] Fornell C., Larcker D.F. (1981). Evaluating structural equation models with unobservable variables and measurement error. J. Mark. Res..

[54] Nunnally J.C., Bernstein I.H. (1994). Psychometric Theory.

[55] Bagozzi R.P., Yi Y. (1988). On the evaluation of structural equation models. J. Acad. Mark. Sci..

[56] Henseler J., Ringle C.M., Sarstedt M. (2015). A new criterion for assessing discriminant validity in variance-based structural equation modeling. J. Acad. Mark. Sci..

[57] Henseler J., Ringle C.M., Sarstedt M. (2016). Testing measurement invariance of composites using partial least squares. Int. Mark. Rev..

[58] Shmueli G., Sarstedt M., Hair J.F., Cheah J.-H., Ting H., Vaithilingam S., Ringle C.M. (2019). Predictive model assessment in PLS-SEM: Guidelines for using PLSpredict. Eur. J. Mark..

[59] Liengaard B.D., Sharma P.N., Hult G.T.M., Jensen M.B., Sarstedt M., Hair J.F., Ringle C.M. (2021). Prediction: Coveted, yet forsaken? Introducing a cross-validated predictive ability test in partial least squares path modeling. Decis. Sci..

[60] Ungureanu B.A., Jităreanu A.F., Ungureanu G., Costuleanu C.L., Ignat G., Prigoreanu I., Leonte E. (2025). Analysis of food purchasing behavior and sustainable consumption in the North-East Region of Romania: A PLS-SEM approach. Sustainability.

[61] Majumder T., Ray N., Chaudhuri S. (2025). The role of marketing strategies and trust in shaping consumer behavior and public health outcomes in the street food sector: A PLS-SEM approach. MDIM J. Manag. Rev. Pract..

[62] Cassidy O., Boyland E., Persky S., Troxel A.B., Elbel B. (2025). Examining the effect of virtual reality–based fast-food marketing on eating-related outcomes in young adults: Protocol for a randomized controlled trial. JMIR Res. Protoc..

